# Utilizing volatile organic compounds for early detection of *Fusarium circinatum*

**DOI:** 10.1038/s41598-022-26078-1

**Published:** 2022-12-15

**Authors:** Ida Nordström, Patrick Sherwood, Björn Bohman, Stephen Woodward, Donnie L. Peterson, Jonatan Niño-Sánchez, Tamara Sánchez-Gómez, Julio Javier Díez, Michelle Cleary

**Affiliations:** 1 Southern Swedish Forest Research Centre, Swedish University of Agricultural Sciences, Box 190, 234 22 Lomma, Sweden; 2 Department of Plant Protection Biology, Swedish University of Agricultural Sciences, Box 102, 234 22 Lomma, Sweden; 3 School of Biological Sciences, Department of Plant and Soil Science, University of Aberdeen, Cruickshank Building, St. Machar Drive, Aberdeen, AB24 3UU UK; 4 iuFOR- Sustainable Forest Management Research Institute, University of Valladolid–INIA, 34004 Palencia, Spain

**Keywords:** Biological techniques, Molecular biology, Plant sciences

## Abstract

*Fusarium circinatum*, a fungal pathogen deadly to many *Pinus* species, can cause significant economic and ecological losses, especially if it were to become more widely established in Europe. Early detection tools with high-throughput capacity can increase our readiness to implement mitigation actions against new incursions. This study sought to develop a disease detection method based on volatile organic compound (VOC) emissions to detect *F. circinatum* on different *Pinus* species. The complete pipeline applied here, entailing gas chromatography—mass spectrometry of VOCs, automated data analysis and machine learning, distinguished diseased from healthy seedlings of *Pinus sylvestris* and *Pinus radiata*. In *P. radiata,* this distinction was possible even before the seedlings became visibly symptomatic, suggesting the possibility for this method to identify latently infected, yet healthy looking plants. *Pinus pinea*, which is known to be relatively resistant to *F. circinatum,* remained asymptomatic and showed no changes in VOCs over 28 days. In a separate analysis of in vitro VOCs collected from different species of *Fusarium*, we showed that even closely related *Fusarium* spp. can be readily distinguished based on their VOC profiles. The results further substantiate the potential for volatilomics to be used for early disease detection and diagnostic recognition.

## Introduction

Forests globally are increasingly threatened by alien invasive pathogens and pests. Globalization is primarily responsible for the increasing rate of establishment of invasive alien species (IAS) and no saturation point is yet predictable^1^. Climate change also compounds the spread of IAS through the elimination of environmental barriers, allowing IAS to establish and survive in new geographic locations. There are many potential pathways of introduction of alien pests and pathogens affecting trees in urban and forested landscapes, *e.g*., trade of plant-derived commodities^2–5^ including seeds^6^, potting substrates and other plant products valuable for other human activities. Preventing new introductions of IAS is achievable through better biosecurity measures at, for example, border entry locations. However, biosecurity in the plant trade is often curtailed by a lack of resources and necessary skills to recognize problems during plant inspections and a lack of modernized tools with high throughput capacity for detection of alien species in plant shipments^7–10^. Countries with stricter border control have fewer established quarantine alien insects^11^ and fungal plant pathogens^12^. According to Santini et al.^2^ approximately 50 invasive forest pathogens currently found in Europe are accidentally introduced alien species, of those approximately 26% attack gymnosperms mainly causing dieback, death and/or reduced growth. Of all invasive forest pathogens in Europe, only 1% have been successfully eradicated by sanitary measures^2^, a likely result of missing the critical window where early detection and rapid response could lead to effective eradication of the founding population.

To combine recent technological advances with knowledge about specific metabolic responses in pests, pathogens and the trees that they infect is a challenge that calls for interdisciplinary competence. Traditional approaches of disease detection are unsatisfactory for largescale plant screening; usually shipments are only spot-checked if at all, and apart from visual scouting for symptoms, testing is generally targeted and uses tedious and expensive DNA-based or serological detection assays^13^. Innovative methods that are better suited for early and rapid detection are needed^13^. Detecting volatile organic compounds (VOCs) released by pathogens and during disease is one such method that could be utilized as an early warning system, facilitating the choice of plant material to be processed for more specific DNA-based diagnosis. As stated by Materić et al*.*^14^*,* VOCs are secondary metabolites produced by all living organisms, the composition of which is unique to every species and presumably also all specific plant-pathogen interactions, comparable to a chemical fingerprint. Emission rates and composition of VOCs are highly dynamic, influenced by biotic and abiotic stresses, and can serve as an indicator of plant health status^14^. Sampling of VOCs can be done in non-destructive ways from many plants simultaneously, and could serve as a high-throughput detection tool for plant diseases^13,15–17^.

Detection of plant pathogens by analysis of VOCs emitted during infection has been reported in multiple studies, for example, for the early detection of spoilage diseases in crops and grains^15,18–22^ and the general understanding is that VOC emissions reflect the specific plant-pathogen combination^23^. Similar methods have also been developed for woody plants with importance for the food industry, like palm^24^ and lemon trees^25^. An example of a VOCs application currently near commercial use is the in-field detection of *Candidatus* Liberibacter asiaticus, a bacterium that is the causal agent of citrus greening disease, commonly known as Huanglongbing, that has devastated the global citrus fruit industry^26,27^. Pathogen detection methods based on VOCs in a forestry context is far less developed. However, Vuorinen et al*.*^28^ could distinguish birch trees exposed to pathogens or herbivores on the basis of VOC profiles, and similarly Johne et al*.*^29^ could differentiate between two pathogenic fungi in horse chestnut trees (*Aesculus* spp.). It has been shown that VOCs may serve as an indicator of fungal infection in asymptomatic spruce^30^ and recently, Brilli et al*.*^31^ found a few VOCs to be uniquely emitted from *Ceratocystis platani*-infected asymptomatic *Platanus* trees, highlighting the potential for targeted VOCs analysis for disease detection. Further method development is needed for VOCs applications in the forestry field.

A method utilizing VOCs requires strategies for collection, separation, detection and analysis of the VOCs. Plant VOC collection is most extensively performed by headspace (HS) sampling, a non-destructive approach offering a more realistic picture of the plant VOC profile as compared to alternatives such as extractions of VOCs from plant tissues in organic solvents. Sampling of HS can be achieved using dynamic methods or by static solid phase micro-extraction (SPME)^16^. SPME fibers are inert and reusable sampling devices having absorbant or adsorbent coatings to which the targeted compounds are sorbed. The chemical properties of the coating determines what type of compounds can be sampled successfully. SPME is easy to use, and once equilibrium with the surrounding HS is reached, the SPME fiber can be thermally desorbed in a gas chromatograph (GC) for subsequent separation of the components in the sample^16^. Analytes separated by GC are most commonly analysed by a flame ionization detector or a mass spectrometer (MS)^15^. The final challenge to complete a detection method pipeline lies in the analysis of the big data sets generated from GC–MS analysis, which can be done utilizing for example MZMine 2, an open-source software for MS data processing^32^. This makes the pipeline fully machine based and, correctly implemented, this approach has potential to be as easily applied as the ion mobility mass spectrometry routinely used in airport security.

*Fusarium* is a large genus of (mostly) plant-associated filamentous fungi, consisting of 23 defined species complexes and almost 300 distinct species^33,34^. *Fusarium circinatum*, the causal agent of pine pitch canker (PPC) disease^35^, poses a serious threat to pine forests across the globe^36^. The *Fusarium fujikuroi* species complex, to which *F. circinatum* belongs, includes several clades of species with a wide plant host range and varying host specificity^37^. The American-clade species *F. circinatum* causes a serious disease on a variety of pine (*Pinus*) species and on Douglas fir (*Pseudotsuga menziesii*)^38^. Early symptoms of *F. circinatum* infection on pine include resinous cankers, chlorosis and/or wilting of needles while late symptoms appear as shoot dieback, reddening and dead foliage^39^. This pathogen originates from the south-eastern USA but has now been recorded in 14 countries across Africa, Asia, South America, and south-western Europe^35,38^. In countries with significant coniferous timber production, preventing the introduction of *F. circinatum* is crucial. Models of the potential spread and damage caused by *F. circinatum* suggest that currently, the pathogen may cause limited damage in pine forests and plantations in Northern Europe, but the potential distribution is expected to expand northward in all climate change scenarios^40^. Even in currently unfavorable geographic regions, *F. circinatum* can thrive in nurseries where it acts as a damping off disease and causes considerable financial consequences also in regions where field conditions are generally not considered suitable for PPC^38^. Plants infected in nurseries will be the origin of future outbreaks in the forest when planted. Once established, *F. circinatum* spreads readily by rain splash, wind and vectoring insects^10,41^ but is also soil-borne^39^. Asymptomatic infection has been reported^42,43^ even in non-pine^44^, grass^45^ and herb species^46^, making visual detection impossible, emphasizing the need for reliable high-throughput diagnostic protocols. Furthermore, *Fusarium* spp. are morphologically very similar, can sometimes be difficult to distinguish by culture morphology, and therefore require more detailed molecular analysis to identify to a species level^37^.

Host susceptibility to PPC varies among pine species. *Pinus radiata* is the most planted conifer globally^47^ and has a large economic and societal value. The species is known to be highly susceptible to PPC and is the main host in northern Spain where PPC is established and causing significant damage^42^*. Pinus sylvestris,* a dominant tree species in northern European forests, and the most widely distributed pine species in the world^48^, is also shown to be susceptible to PPC based on greenhouse and field inoculation trials on young trees of Spanish, Scottish and Czech origin^38,44,49^. *Pinus pinea* is distributed all around the Mediterranean basin including northern Spain, which could enable rapid spread of this plant disease. However, *P. pinea* has remarkable phenotypic plasticity in functional traits that may explain its relatively higher resistance to *F. circinatum*-infection^50^ compared to other pine species.

The aim of this study was to develop a disease detection method based on VOC emissions from pine seedlings. By establishing a library of chemical fingerprints characterizing specific emission profiles, it should prove possible to non-destructively scan plant consignments in ports of entry or plant nurseries to detect the presence of disease, and rapidly respond with further measures to limit its establishment and potential losses. The study sought to: (1) test whether in vitro VOC signatures can distinguish between different *Fusarium* spp. (2) examine whether in vitro* F. circinatum* VOCs are present in in vivo, and (3) test whether infection of *F. circinatum* on pine seedlings can be detected on the basis of VOCs prior to expression of visible symptoms of disease.

## Results and discussion

### *Fusarium* spp. cultured on defined media are readily distinguished by VOC profiles

**Figure 1:**
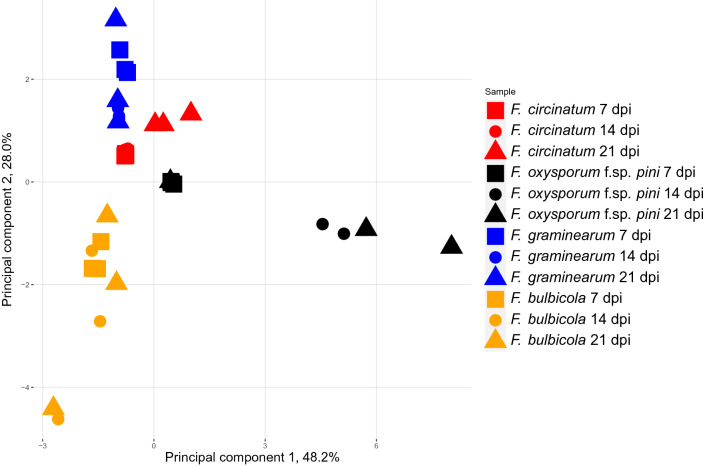
Principal component analysis (PCA) of VOCs emitted by four Fusarium spp. The PCA was computed based on a subset of the 11 VOCs identified by ten consecutive runs of Randomforest. n = 3, each of which were repeatedly sampled at three time points; 7, 14 and 21 days post inoculation (dpi).

As an initial proof-of-concept pilot study, VOC profiles of four *Fusarium* spp. (*F. circinatum*, *F. oxysporum* f.sp. *pini*, *F. bulbicola* and *F. graminearum*) grown in vitro were compared to test whether analysis of VOCs alone could distinguish closely related species. The selection of the three *Fusarium* spp. included here in addition to *F. circinatum* was based on their genetic proximity to *F. circinatum*^51,52^. The VOCs were collected using SPME, analyzed by GC–MS before the output data was processed through an objective pipeline. Several combinations consisting of 3–6 VOCs fulfilled the criteria to distinguish the four species with a significant accuracy (p ≤ 0.05) for every pairwise interspecific comparison, explained further below. An example of a VOC combination utilizable to distinguish the *Fusarium* spp. regardless of timepoint, *i.e.* 7–21 days post inoculation (dpi), with a 0% confusion matrix error rate and p = 0.006, is shown in Table 1. A total of 211 different VOCs were detected from the four *Fusarium* spp. A visualized principal component analysis (PCA) of the *Fusarium* spp. separation based on the 11 VOCs identified by ten repeated Randomforest runs demonstrated the unambiguous groupings irrespective of time point (Fig. 1). A larger study with more replicates would be required to draw confident conclusions regarding the VOC emission characteristics by each species. The results presented here do point to the potential for VOCs analysis as a novel fungus identification method to replace current inadequate or challenging morphological or time-consuming DNA-based approaches that often fall short due to the high morphological and genetic similarity of these species.

**Table 1:** Randomforest selection of VOCs to distinguish between Fusarium species.

Retention time (min)	Retention index (RI)	Tentative ID	F. circinatum*	*F. oxysporum* f.sp. pini*	F. bulbicola*	F. graminearum*
25.34	1417	Unknown sesquiterpene	1.85 ± 0.983	103 ± 24.7	ND	ND
27.09	1515	Unknown oxygenated sesquiterpene 1	ND	391 ± 138	223 ± 67.4	ND
30.24	1778	Unknown oxygenated sesquiterpene 2	7.00 ± 0.745	ND	ND	ND

PERMANOVA analysis with pairwise interspecific comparisons based on three VOCs, selected by Randomforest from MZMine 2-processed data, resulted in significant differences (p = 0.006). Tentative compound IDs for the VOCs and their respective retention indices are given, as well as average base peak area ± standard error (n = 3) for each VOC and species. ND indicates that no peaks above the applied threshold were detected.

*All values are to be multiplied by 10^4^.

The Randomforest selected VOCs observed in Table 1 could not be identified further than to chemical classes, as none of their respective MS data matched any compound in the MS databases, see methods. All three of the compounds were, however, sesquiterpenes, a chemical class previously reported to be emitted from species in the *Fusarium fujikuroi* species complex^53^. It is known that plant emitted monoterpenes such as limonene and linalool can inhibit germination of fungal spores^54,55^, which makes it interesting to find that hyphae of plant pathogenic fungi emit similar compounds, such as the sesquiterpenes found here, emitted by *Fusarium* spp. There were a number of compounds found to be exclusively detected in just one of the four *Fusarium* spp. despite the close genetic proximity of the species, for example oxygenated sesquiterpene 2 exclusively emitted by *F. circinatum* (Table 1). This finding demonstrated the ease with which a VOCs-based detection method could distinguish between morphologically and genetically similar *Fusarium* spp.

### VOC profiles can distinguish between *F. circinatum*-inoculated and mock-inoculated seedlings

**Figure 2:**
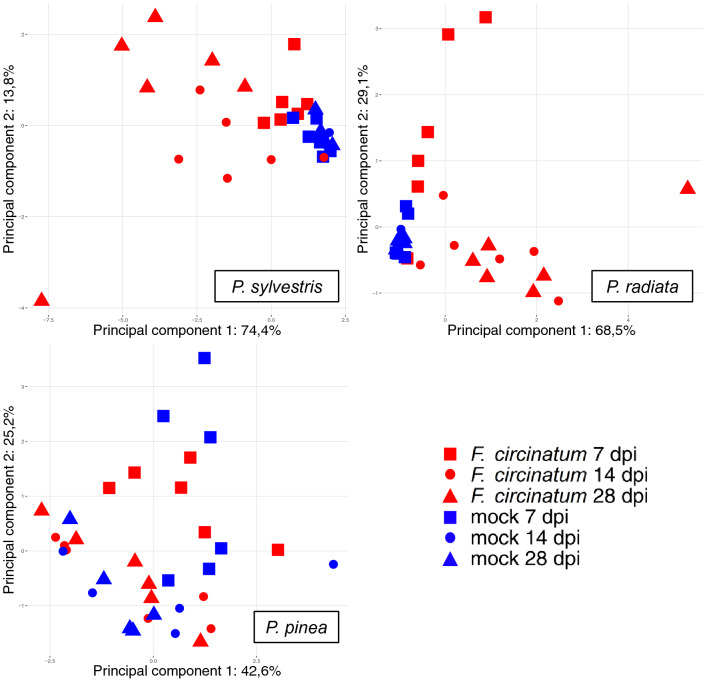
Principal component analysis of VOC subsets from *Pinus* spp. seedlings inoculated with *F. circinatum* or mock inoculated. n = 5–6 seedlings per inoculation type, sampled at three time points: 7, 14 and 28 days post inoculation (dpi). Percentages given on each axis of the plots show the total variance explained by that principal component.

VOCs were sampled from stem-inoculated seedlings of *P. sylvestris, P. radiata* and *P. pinea*. *Fusarium circinatum-*inoculated seedlings were compared to control (mock-inoculated) seedlings, hereafter referred to as “inoculation types”, at 7, 14 and 28 dpi. The same pipeline used for the in vitro studied *Fusarium* spp. was applied to these in vivo samples, including MZMine 2, Randomforest and PERMANOVA for GC–MS data analysis, which resulted in a number of significant distinctions (Table 2). There were significant (p ≤ 0.05) differences in VOC profiles between the two different inoculation types of *P. radiata* at all time points, including the earliest time point at 7 dpi when no symptoms were yet visible. In terms of detection tool development for biosecurity, the ability to detect disease earlier than the point of symptom appearance is an important detail. This enables identification of infected, yet apparently healthy seedlings that could otherwise slip through ports of entry and plant nurseries unnoticed, an introduction pathway that remains difficult to address. For *P. sylvestris,* significant differences were seen at 14 and 28 dpi, and for *P. pinea,* considered to have very low susceptibility to *F. circinatum*, no symptoms developed and no significant differences in VOCs emissions were observed between the inoculation types at any time point. These results are visualized by a principal component analysis (Fig. 2). None of the VOCs detected were exclusively detected in the *F. circinatum* inoculated seedlings, therefore the analysis was based on relative quantitative comparisons between samples.

**Table 2:** Summary of PERMANOVA comparison p-values of *Pinus* seedlings.

Time point	*P. sylvestris*	*P. radiata*	*P. pinea*
7 dpi	0.071	**0.015**	0.537
14 dpi	**0.003**	**0.007**	0.839
28 dpi	**0.003**	**0.043**	0.465

P-values represent intraspecific differences between *F. circinatum*- and mock- inoculated seedlings at each time point; significant p-values (p ≤ 0.05) in bold. VOCs were recorded at three time points; 7, 14 and 28 days post inoculation (dpi), n = 5–6.

Randomforest produced a subset of eight compounds for *P. sylvestris,* three for *P. radiata* and five for *P. pinea*, which were subsequently utilized in the statistical models (Table 3). These compounds were detected through machine learning, within the complete VOC profiles, as important because of their low error rate as indicators of the seedling inoculation type irrespective of time point (see methods). A total of 307 unique VOCs were found between the three *Pinus* spp., most of which were present in all three species. It is possible that VOCs other than the subset found here by Randomforest could strengthen the outcomes, for example the distinction between *P. sylvestris* inoculation types specifically at 7 dpi, if Randomforest had been set to examine each timepoint separately. However, the objective here was to find VOCs that allow for a robust distinction irrespective of time post infection, as a detection method must be applicable regardless of the (often unknown) infection age.

**Table 3:** Tentative IDs of VOCs identified by Randomforest as suitable indicators for distinguishing between mock- and *F. circinatum*-inoculated pine seedlings.

Species	Retention time	RI	RI ref	Tentative ID
*P. sylvestris*	12.15	958	952^1^	camphene
	12.26	963	957^2^	verbenene
	14.13	1032	1026^2^	*p-*cymene
	15.98	1098	–	propenyl toluene isomer
	17.74	1155	1143^2^	trans-verbenol
	18.78	1190	1173^2^	isopinocamphone
	19.46	1212	1212^3^	homomyrtenol
	19.85	1224	1204^2^	verbenone
*P. radiata*	12.26	963	957^2^	verbenene
	15.98	1098	–	propenyl toluene isomer
	19.85	1224	1204^2^	verbenone
*P. pinea*	11.72	941	939^2^	α-pinene
	14.86	1058	–	unknown
	16.52	1115	–	spiro[4,5]decane
	25.34	1416	–	dimethoxy-propenyl benzene isomer
	26.54	1479	–	unknown

^1^:^56^ ^2^:^57^ ^3^:^58^.

RI = retention index.

**Figure 3:**
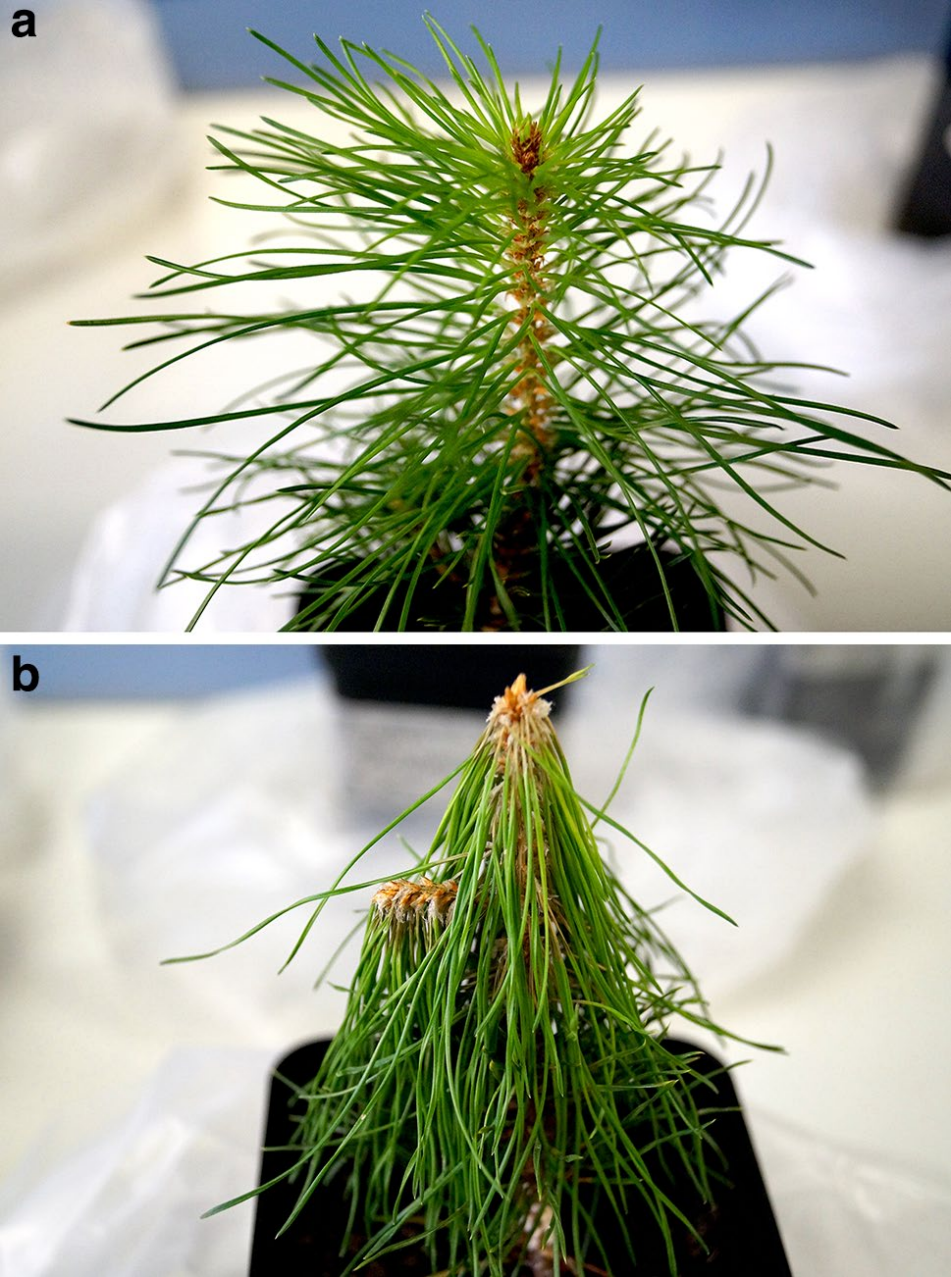
Symptom development in *P. sylvestris* shoots following stem inoculations. (**a**) Mock inoculated *P. sylvestris* seedling at 28 dpi, a healthy shoot with no signs of disease; (**b**) *Fusarium circinatum* inoculated *P. sylvestris* seedling at 28 dpi, displaying characteristic symptoms of shoot wilting and needle chlorosis.

Observations of symptom development were carefully documented throughout the experiment. All *Pinus* spp. had resinous wounds at the inoculation site at 7 dpi, but were otherwise asymptomatic at this time, regardless of inoculation type. *Fusarium circinatum-*inoculated *P. sylvestris* and *P. radiata* were consistently symptomatic at 14 dpi, with light chlorosis and/or slight wilting of needles, which was described as grade 1 symptoms in the scale used by Martín-Rodrigues et al.^39^. At 28 dpi, symptoms on *P. sylvestris* and *P. radiata* had progressed to grade 3, with severe wilting (Fig. 3). *Pinus pinea* seedlings remained asymptomatic at all time points. Symptom development on *P. sylvestris* and *P. radiata* were consistent with previous reports of inoculations on 2-year-old *P. radiata*^59^. The *P. sylvestris* used in this study had similar susceptibility as *P. radiata* to *F. circinatum*, underlining the potentially serious threat posed by PPC to forests of northern Europe dominated by *P. sylvestris*.

No VOCs detected were uniquely, and consistently, emitted from *F. circinatum-*inoculated seedlings. Therefore, no single VOC detected here can independently be used as a reliable indicator of disease, which also rules out the idea of identifying a *F. circinatum*-specific VOC emitted regardless of growth medium, as could be done for example in the study by Brilli et al.^31^. This means that multivariate data analysis, preferably using a machine learning-based pipeline as presented in this study, is required.

The use of a fully automatic pipeline entailing automated data analysis and machine learning instead of manual processing, beyond being immensely time saving, eliminates the risks of introducing human errors, arbitrariness and need for GC–MS expertise. Advanced competence is needed to manually process GC–MS data, identifying a few hundred VOCs per run sample, and peaks (corresponding to VOCs in the samples) often coincide, making manual peak integration impossible. By elimination of manual processing, the detection method can be performed by nonexperts, overcoming barriers of entry to use this kind of detection method. Machine learning can, in addition, allow for detection of multivariate patterns that are difficult or impossible to detect manually, increasing the detection accuracy. Its accuracy can be further improved by calibrating the models using much bigger data sets than for example the ones available in this study.

The VOCs identified as predictors of the *Pinus* seedling inoculation type were tentatively structurally identified based on retention indices and comparison with mass spectral data from libraries and previous literature (Table 3). These VOCs were predominantly terpenoids, chemicals strongly associated with VOC emission from pine trees. One example is verbenone, a monoterpene found important in the distinction of mock- and *F. circinatum-*inoculated *P. sylvestris* as well as *P. radiata*, as emission levels were higher in infected trees. Verbenone is known to be emitted from a variety of plants, and also functions as an insect pheromone with important roles, for example, as a repellent to mountain pine beetles^60^. A list of the Randomforest-selected VOCs and some of their known functions is found in the supplementary information (Table S1).

Our study showed that VOCs analysis can distinguish *F. circinatum*-infected *P. radiata* seedlings before visible symptom development, suggesting the potential to scale up this detection tool for in-field use. In this study, a benchtop GC–MS instrument was used, but other options include the electronic nose (E-nose) and portable GC–MS instruments. In contrast to the E-nose that only detects specifically targeted VOCs classes, GC–MS theoretically can detect all VOCs present in a sample. Additionally, the E-nose detection limits are typically in the μg L^−1^ range, as compared to pg-ng for FID and MS^15^, suggesting that the portable GC–MS may be a better option for use in an up-scaled and in-field scenario. When combined with SPME HS sampling, it has potential as an application for high-throughput detection of problems in large plant shipments. Portable GC–MS instruments are commercially available, and for example are currently employed by Homeland Security in the U.S., with similar sensitivity to a basic benchtop instrument^61^. Portable GC–MS has been used to distinguish between healthy and pest-infested milkweed (*Asclepias* spp.)^62^ as well as to readily identify potential fungal biomarkers when coupled with SPME^63^. This would make an interesting alternative for testing in future work with forest pathogens, especially *F. circinatum* based on our results, for detecting the pathogen in asymptomatic seedlings in nursery consignments, but also in soil, another known pathway of introduction for this pathogen.

Comparing the VOCs profiles of pine seedlings inoculated with different pine pathogens would be an important next step to this work. Such a comparison could determine whether VOCs profiles of pine seedlings inoculated with different pathogens can be distinguished from one another (as described in horse chestnut trees by Johne et al*.*^29^), or whether pine seedlings’ VOCs responses to fungal pathogens are non-specific, yet further investigations are warranted.

## Methods

### *Fusarium* spp. cultured on defined media

For examination and comparison of VOCs produced by *Fusarium*, four *Fusarium* spp. were grown on defined Elliott’s medium agar (EMA) without sterol^64^: *Fusarium circinatum*, the closely related *F. bulbicola*, the intermediately related *F. oxysporum* f.sp. *pini*^51^ and the more distantly related *F. graminearum*^52^. For strain information, see Table S2. EMA was dispensed in slanted 20 mL clear glass vials (Merck KGaA, Darmstadt, Germany), capped with permeable magnetic screw caps with polytetrafluoreten/silicone 1.33 mm septa (Merck KGaA, Darmstadt, Germany). The capped vials were incubated at room temperature under natural light conditions and sampled for 24 h at days 7, 14 and 21 after sub-culture by inserting divinylbenzene/carboxen/polydimethylsiloxane SPME fibers through the septa. The SPME needle size was 24 ga, 2 cm long and coated with 30 μm (CAR/PDMS layer), 50 μm (DVB layer) (Merck KGaA, Darmstadt, Germany).

### *Fusarium circinatum* inoculated *Pinus* spp

*Fusarium circinatum* strain *FcCa6* (obtained from the laboratory of Prof. Julio Javier Díez) was stem-inoculated on 1 year-old *P. sylvestris, P. radiata* and *P. pinea* (for information on sources, see Table S3). The seedlings were obtained from Viveros y Servicios Forestales Caselas, S.L., a nursery in Mondoñedo Lugo, Spain, and transported by express courier in December 2020 to the forest pathology laboratory of the Universidad de Valladolid, Palencia, Spain. The plant material used in this study complies to relevant guidelines and all necessary permissions were in place. Seedlings were transplanted to 0.77 L pots into black peat moss previously autoclaved twice at 121 °C for 20 min. Plants were acclimated for 3 months in a climate chamber at 21.5 °C under a 16/8 h day/night regime and approximately 68% relative humidity. Throughout the acclimation and experimental period, seedlings were watered twice a week.

Stem inoculations were performed, using a method described elsewhere^59,65^, by cutting a small wound on the stem, approximately 7 cm above the root collar and applying 20 µL of a potato dextrose broth (PDB) (Sharlau Microbiology, Barcelona, Spain) based spore suspension containing 10^6^ *F. circinatum* spores mL^-1^, directly to the surface of the wound. Wounds were covered with Parafilm (Bemis Company Inc., Neenah, USA) until the start of the SPME sampling. Mock inoculations were identical but without spores in the PDB. During the experiment, symptom development was observed and documented on the seedlings. To confirm that the mycelial growth seen on stems was *F. circinatum*, the mycelia were harvested and sub-cultured to EMA^64^ before examination under the microscope, where coiled sterile hyphae characteristic of *F. circinatum* were seen. VOCs sampling was performed using SPME fibers for static HS sampling: each seedling, including pot, and SPME fiber was wrapped in 38 L high-density polyethylene bags (Labbox labware, Barcelona, Spain), maintained at room temperature for 24 h and thereafter the SPME samples were immediately analyzed using GC–MS.

### GC–MS and data analysis

Immediately after sampling, the SPME fibers were manually injected through an ultra-inert, splitless, straight, 2 mm liner (Agilent, Santa Clara, USA) on a 6890 N GC (Agilent Technologies, Santa Clara, USA) coupled with a 5973 MS (Agilent Technologies, Santa Clara, USA). The column was a HP-5 ms ultra inert 60 m GC column, 0.25 mm, 0.25 µm, 7 inch cage (Agilent, Santa Clara, USA). A C8-C20 hexane mix (Merck KGaA, Darmstadt, Germany) was used as an assurance that there was no shift in retention time over the project time span. GC–MS was performed through MSD ChemStation version E.02.02.1431 (Agilent Technologies, Santa Clara, USA) with an initial oven temperature of 50 °C, followed by an 8 °C/min increase to 100 °C, subsequently increasing by 4 °C/min to 160 °C, a final ramp of 16 °C/min to 280 °C and hold for 2.5 min (Table S4). GC–MS data were transformed to .cdf files and processed (ADAP chromatogram builder, chromatogram deconvolution, multivariate curve resolution) and aligned (ADAP aligner) with MZMine 2 (v 2.53)^32^. The Randomforest compound selection (see below) for distinguishing between mock- or *F. circinatum-*inoculated seedlings (in vivo), or *Fusarium* species (in vitro), were tentatively identified by matching mass spectrometry data and back-calculated retention indices^66^ with literature values from authentic standards found in Nist20 and Wiley12 MS databases.

### Programming, machine learning and statistical tests

Randomforest and VarSelRF^67^ are two packages in R^68^ that were used to select a reduced model to a set of VOCs that were predictive of *Fusarium* spp. in the in vitro and inoculation type in the in vivo experiments^69^. Randomforest is used to tune and reduce the model error and VarSelRF chooses a model of VOCs with the lowest error rate. VarSelRF uses the confusion matrix testing parameter “out-of-bag” error as a criterion to remove variables (*i.e.* individual VOCs) in a backward elimination starting with the least important VOCs. The least important variables are those defined by Randomforest from the mean decrease in accuracy^70^. The model selection stops when current out-of-bag error rate becomes larger than the previous iteration. The selected subsets of *Fusarium* spp. and *Pinus* spp. VOCs were then run through Permutational Multivariate Analysis of Variance (PERMANOVA)^71^, to determine relative differences in VOCs between *Fusarium* spp., or inoculation type in *Pinus* spp. Posthoc Holm tests^72^ were thereafter applied. Data for PERMANOVA were Hellinger transformed. The Stats package, prcomp function was used to generate the PCA analysis and plot, scaling the input data to visually display differences among the compared groups (RStudio, version 1.1.456).

### Additional information

All necessary permissions were obtained to complete this study, no ethics considerations are applicable. Supplementary data is publicly available through the Swedish National Data Service (SND), doi: 10.5878/hc9w-7694. The voucher specimens of the three *Pinus* species included in this study were provided by Viveros y Servicios Forestales Caselas, S.L., a nursery in Mondoñedo Lugo, Spain, but have not been deposited to any publicly available herbarium. The *Fusarium circinatum* isolate *FcCa6* used in this study was identified in previous work by Martínez-Álvarez^73^, provided and maintained by the laboratory of Prof. Díez, available in lab collections in several countries but yet no public herbarium.

## Supplementary Information

Supplementary Information 1.

Supplementary Information 2.

Supplementary Information 3.

Supplementary Information 4.

Supplementary Information 5.

## Data Availability

The data that support the findings of this study are openly available in doi: 10.5878/hc9w-7694.
