# Supplementary material for: Utilizing volatile organic compounds for early detection of Fusarium circinatum
Source: Sci Rep. 2022 Dec 15;12:21661. doi: 10.1038/s41598-022-26078-1 (PMC9755288; doi:10.1038/s41598-022-26078-1)
Supplement: Supplementary file 1 — Supplementary Information 1. [file 41598_2022_26078_MOESM1_ESM.pdf]

Supplementary information

Table S1 | Randomforest-selected VOCs as indicators of *F. circinatum*-infection in *Pinus* seedlings and examples of their known functions.

Tentative ID	Examples of identified functions
camphene	Monoterpene common in biogenic VOC blends emitted from for example Scots pine stands (Bäck *et al.*, 2012; Komenda & Koppmann, 2002) and nest mounds (Šimpraga *et al.*, 2019). A minor constituent of many essential oils (Pino *et al.*, 2005).
verbenene	Monoterpene that can act as an aggregation pheromone for spruce beetles (Gries *et al.*, 1992). Has been found in honey (Karabagias *et al.*, 2020)
p-cymene	Alkylbenzene, related to a monoterpene, released from foliage and flowers (Šimpraga *et al.*, 2019), for example Scots pine (Bäck *et al.*, 2012)
propenyl toluene isomer	Unknown
trans-verbenol	Monoterpene alcohol and mountain pine beetle pheromone, attracting insects (Chiu *et al.*, 2018).
isopinocamphone	Monoterpenoid emitted by plants, for example Hyssop (Ahmadi *et al.*, 2020), and a compound that generated great response in the electroantennogram of a larval parasitoid of the southern pine beetle (Salom *et al.*, 1992)
homomyrtenol	Terpene emitted from plants, for example by carrots, frequently used in chemical applications to modify materials (Hu *et al.*, 2022).
verbenone	Monoterpene emitted from many plants, and also functions as an insect pheromone with important roles, for example, as a repellent to mountain pine beetles (Fettig & Munson, 2020; Rappaport *et al.*, 2001)
α-pinene	One of the most abundant monoterpenes in biogenic VOC blends from Scots pine stands (Bäck *et al.*, 2012; Komenda & Koppmann, 2002; Shao *et al.*, 2001). Attracts spruce beetles (Gries *et al.*, 1992).
spiro[4,5]decane	Unknown
dimethoxy-propenyl benzene isomer	Unknown

Table S2 | Information on *Fusarium* species used in this study.

Species complex	***Species***	***Strain***	***Source***
F. fujikuroi	*F. circinatum*	*FcCa6**	Escuela Técnica Superior de Ingenierías Agrarias, Universidad de Valladolid, Palencia, Spain
F. fujikuroi	*F. bulbicola*	NRRL 13618, CBS: 220.76	Westerdijk Fungal Biodiversity Institute, Utrecht, Netherlands
F. oxysporum	*F. oxysporum* f.sp. *pini*	NRRL 22551, CBS: 171.31	Westerdijk Fungal Biodiversity Institute, Utrecht, Netherlands
F. graminearum	*F. graminearum*	---	Dept. Plant Breeding, Swedish University of Agricultural Sciences, Alnarp, Sweden

*virulence previously described by Martínez-Álvarez *et al.* (2012).

Table S3 | Sources of *Pinus* seedlings used in this study. All seedlings were approximately one year old by the start of the experiments.

**Species**	**Source**
Pinus sylvestris	ES10: Sierra de Guadarrama. Seed orchard HS-Valsaín-1
Pinus radiata	Mountain and interior plateau of Galicia, Spain
Pinus pinea	ES01: North Plateau

Table S4 | GC-MS method. The settings used for a 6890N GC machine (Agilent Technologies, Santa Clara, USA) coupled with a 5973 MS (Agilent Technologies, Santa Clara, USA), using MSD ChemStation software version E.02.02.1431 (Agilent Technologies, Santa Clara, USA).

**Oven**	**Rate (°C/min)**	**Value (°C)**	**Hold time (min)**	**Run time (min)**
**Initial**		50	2	2
**Ramp 1**	8	100	0	8.25
**Ramp 2**	4	160	0	23.25
**Ramp 3**	16	280	2.5	33.25

Supplementary data

See separate excel sheet for VOCs data. The peaks are sorted and comparable by column, named after their retention times.

References

Ahmadi, H., Babalar, M., Sarcheshmeh, M.A.A., Morshedloo, M.R. & Shokrpour, M. (2020). Effects of exogenous application of citrulline on prolonged water stress damages in hyssop (Hyssopus officinalis L.): Antioxidant activity, biochemical indices, and essential oils profile. *Food Chemistry,* 333, p. 127433.

Bäck, J., Aalto, J., Henriksson, M., Hakola, H., He, Q. & Boy, M. (2012). Chemodiversity of a Scots pine stand and implications for terpene air concentrations. *Biogeosciences,* 9(2), pp. 689-702.

Chiu, C.C., Keeling, C.I. & Bohlmann, J. (2018). Monoterpenyl esters in juvenile mountain pine beetle and sex-specific release of the aggregation pheromone <i>trans</i>-verbenol. *Proceedings of the National Academy of Sciences,* 115(14), pp. 3652-3657.

Fettig, C.J. & Munson, A.S. (2020). Efficacy of verbenone and a blend of verbenone and nonhost volatiles for protecting lodgepole pine from mountain pine beetle (Coleoptera: Curculionidae). *Agricultural and Forest Entomology,* 22(4), pp. 373-378.

Gries, G., Borden, J.H., Gries, R., Lafontaine, J.P., Dixon, E.A., Wieser, H. & Whitehead, A.T. (1992). 4-Methylene-6,6-dimethylbicyclo[3.1.1]hept-2-ene (verbenene): New aggregation pheromone of the scolytid beetleDendroctonus rufipennis. *Naturwissenschaften,* 79(8), pp. 367-368.

Hu, Y., Yang, Z., Gao, Y., Nie, J. & Sun, F. (2022). Synthesis and properties of bio-based cationic photopolymerizable polysiloxane oxacyclobutanes based on nopol. *Progress in Organic Coatings,* 163, p. 106681.

Karabagias, I.K., Maia, M., Karabournioti, S., Gatzias, I., Karabagias, V.K. & Badeka, A.V. (2020). Palynological, physicochemical, biochemical and aroma fingerprints of two rare honey types. *European Food Research and Technology,* 246(9), pp. 1725-1739.

Komenda, M. & Koppmann, R. (2002). Monoterpene emissions from Scots pine (Pinus sylvestris): Field studies of emission rate variabilities. *Journal of Geophysical Research: Atmospheres,* 107(D13), pp. ACH 1-1-ACH 1-13.

Martínez-Álvarez, P., Alves-Santos, F.M., Diez, J.J. (2012). In vitro and in vivo interactions between Trichoderma viride and Fusarium circinatum. *Silva Fennica,* 46(3).

Pino, J.A., Mesa, J., Muñoz, Y., Martí, M.P. & Marbot, R. (2005). Volatile components from mango (Mangifera indica L.) cultivars. *J Agric Food Chem,* 53(6), pp. 2213-23.

Rappaport, N.G., Owen, D.R. & Stein, J.D. (2001). Interruption of Semiochemical-Mediated Attraction of Dendroctonus valens (Coleoptera: Scolytidae) and Selected Nontarget Insects by Verbenone. *Environmental Entomology,* 30(5), pp. 837-841.

Salom, S.M., Ascoli-Christensen, A., Birgersson, G., Payne, T.L. & Berisford, C.W. (1992). Electroantennogram responses of the southern pine beetle parasitoid Coeloides pissodis (Ashmead) (Hym., Braconidae) to potential semiochemicals. *Journal of Applied Entomology,* 114(1-5), pp. 472-479.

Shao, M., Czapiewski, K.V., Heiden, A.C., Kobel, K., Komenda, M., Koppmann, R. & Wildt, J. (2001). Volatile organic compound emissions from Scots pine: Mechanisms and description by algorithms. *Journal of Geophysical Research: Atmospheres,* 106(D17), pp. 20483-20491.

Šimpraga, M., Ghimire, R.P., Van Der Straeten, D., Blande, J.D., Kasurinen, A., Sorvari, J., Holopainen, T., Adriaenssens, S., Holopainen, J.K. & Kivimäenpää, M. (2019). Unravelling the functions of biogenic volatiles in boreal and temperate forest ecosystems. *European Journal of Forest Research,* 138(5), pp. 763-787.
